# Experiences pertaining to child nutrition and care provision among early care and education stakeholders, sponsors, and center directors during the COVID-19 pandemic: A multi-method study

**DOI:** 10.3389/fpubh.2022.999272

**Published:** 2022-12-08

**Authors:** Temitope Erinosho, Bethany Jana, Kaitlyn Loefstedt, Maihan Vu, Dianne Ward

**Affiliations:** ^1^Department of Applied Health Science, Indiana University Bloomington, Bloomington, IN, United States; ^2^Department of Health Behavior and the Center for Health Promotion and Disease Prevention, University of North Carolina at Chapel Hill, Chapel Hill, NC, United States; ^3^Department of Nutrition and the Center for Health Promotion and Disease Prevention, University of North Carolina at Chapel Hill, Chapel Hill, NC, United States

**Keywords:** early care and education, child care, nutrition, child and adult care food program, COVID-19 pandemic, multi-method study

## Abstract

**Purpose:**

This study used multiple methods (interviews, survey) to assess experiences of stakeholders, sponsors, and center-based early care and education (ECE) program directors pertaining to child nutrition (e.g., provision of nutritious foods, mealtime practices, CACFP administration/use) and the provision of child-care (i.e., day-to-day ECE operations and programming) during the COVID-19 pandemic.

**Methods:**

Participants included stakeholders from 22 national and state agencies associated with the Child and Adult Care Food Program (CACFP) who also work to promote nutrition and quality child-care, representatives of 17 CACFP sponsor organizations, and 40 center-based ECE program directors who participated in interviews, as well as 100 ECE directors who completed surveys. Data were collected across four states. Thematic analyses of interviews and descriptive methods were used to analyze data collected.

**Results:**

Six main themes emerged from stakeholders, sponsors, and ECE program directors' focusing on: experiences during the temporary closure of several ECE programs; additional responsibilities and unanticipated expenses for ECE programs; difficulty in keeping up with constantly changing COVID-19 guidance; encounters during shifts from in-person to virtual training and monitoring; changes to nutrition practices at ECE; and the need to prioritize ECE funding.

**Conclusions:**

Findings highlight challenges and supports to ECE programs and could inform future efforts to enhance child-care quality and child nutrition in the U.S. during pandemic situations.

## Introduction

One in seven U.S. children experience poverty (1), which puts them at risk for poor nutrition (2). With nearly two-thirds (62%) of children under the age of 6 attending early care and education (ECE) programs for at least 27 h/week (3, 4), ECE is an important setting in which to implement programs to reduce poverty-related health disparities, including food insecurity (4, 5). Since 2020, the coronavirus (COVID-19) pandemic has impacted everyday life (6), including ECE programming. Early in the pandemic, several states instituted stay-at-home orders and mandated ECE programs to shut-down or severely limit child enrollment to prevent COVID-19 transmission (7). Recognizing the risk for harm due to the loss of ECE-provided meals to low-income children, the Families First Coronavirus Response Act, signed into law on March 18, 2020, authorized the U.S. Department of Agriculture (USDA) to offer waivers for the Child and Adult Care Food Program (CACFP), a federally-funded program that reimburses ECE for serving nutritious meals to low-income children (7, 8). The CACFP waivers allowed for non-congregate feeding, including the offering of meals off-site through “grab and go,” and delivery; permitted parental pick-up of non-congregate meals; provided simplified food procurement rules; and allowed for specific meal pattern modifications when certain foods were not available (9, 10). With the waivers, state agencies (stakeholders) and CACFP sponsors could conduct virtual audits and monitoring visits to assess CACFP compliance at ECE (9, 10). While a few studies have surveyed ECE providers to learn about their experiences and strategies for connecting families with food during COVID-19 (6, 11, 12), little is known about experiences of stakeholders and sponsors. Using interviews and a survey, this multi-method study describes experiences of stakeholders, sponsors, and center-based ECE program directors pertaining to child nutrition (e.g., provision of nutritious foods, mealtime practices, CACFP administration/use) and the provision of child-care (i.e., day-do-day ECE operations and programming) during COVID-19. We defined stakeholders as national/state representatives of CACFP and other agencies that work with ECE to promote child nutrition and quality care, whereas sponsors were public/private non-profit organizations that took on administrative responsibilities of operating CACFP on behalf of ECE programs (13). Stakeholders and sponsors play important roles in promoting child nutrition at ECE, hence the reason for including them in this study. Conducting interviews allowed the study team to explore experiences shared by participants, that then guided the development of a survey to assess prevalence with which experiences were reported.

## Methods

### Participants and setting

This descriptive study was conducted across four states—Arizona, North Carolina, New York, and Texas—from December 2020 to November 2021, as part of a larger study to assess barriers and facilitators of ECE participation in CACFP (revise and resubmit, under review). Given the broader focus on CACFP, the criteria for selecting states included: having varying levels of CACFP participation; child poverty levels above the national average; and convenience of data collection. A threshold of 50% defined low- vs. high-CACFP participation by ECE programs. Four states with varying participation levels were selected: Arizona and North Carolina, with 35 (804/2,237) and 49% (2,289/4,642) CACFP participation, respectively, were categorized as low CACFP participation states, whereas New York (4,079/5,856) and Texas (6,753/9,612), both with 70% CACFP participation, were categorized as having higher participation (14–16). All four states exceeded the national child poverty rate (17), and were states in which the study team had existing collaborations with partners at ECE agencies that would help to facilitate data collection.

Center-based ECE programs can participate in CACFP independently by working directly with their state agency (stakeholder), or they can participate through a sponsor that takes on administrative responsibilities of operating CACFP (13). In this study, participants in each state included stakeholders, sponsors, and center-based ECE program directors. Stakeholders included representatives of CACFP and other agencies that work with ECE to promote child nutrition and quality care (e.g., ECE licensing, Child Care Resource and Referral, Quality Rating and Improvement Systems). Potential stakeholders were identified from their agency's websites, while sponsors were identified from lists obtained from the National CACFP Sponsors' Association (18) and state ECE licensing offices. Recruitment strategies included telephone calls and/or email, word of mouth, and recommendations from stakeholders/sponsors.

To identify center-based ECE program directors (“directors” hereon), the study team obtained electronic lists of CACFP and non-CACFP programs from each state's CACFP and ECE licensing agency. Given the broader focus on CACFP barriers/facilitators, a propensity score procedure was used to match CACFP programs in each state with a non-CACFP counterpart in a ZIP Code with similar rural vs. urban classifications (19), similar poverty levels, and household income levels below the state's median income. A random sample of ECE programs in CACFP and potentially-eligible non-CACFP programs was selected, excluding Head Start, Tribal, and school-based programs. Two separate sample pools of ECE programs were drawn for interviews vs. surveys. Telephone calls and a screener were used to determine eligibility and invite directors to participate, with a goal to recruit CACFP/non-CACFP programs in a 3:2 ratio and rural/urban programs in a 1:1 ratio.

Stakeholders from 22 CACFP-associated agencies, representatives of 17 sponsor organizations, and 140 ECE directors participated in this study (Table 1). The study was approved by the Institutional Review Board at Indiana University Bloomington and University of North Carolina at Chapel Hill. Before data collection, participants received an informed consent document. Persons who participated in interviews gave verbal consent. For survey participants, continuing past the online informed consent page implied consent.

**Table 1:** Description of stakeholders, sponsors, and center-based early care and education (ECE) program directors that participated in interviews and surveys.

	**Arizona**	**North Carolina**	**New York**	**Texas**	**National**	**Total**
**Interview participants (*****n*** **= 79)**^d^						
**Stakeholders** ^a^	5	5	5	6	1	22
**Sponsors** ^b^	3	5	4	5	n/a	17
**ECE directors** ^c^	11	8	11	10	n/a	40
*CACFP programs*	5	6	6	6	n/a	23
*Non-CACFP programs*	6	2	5	4	n/a	17
**Survey participants (*****n*** **= 100)**^d^						
**ECE directors** ^c^	24	23	27	26	n/a	100
*CACFP programs*	12	21	19	17	n/a	69
*Non-CACFP programs*	12	2	8	9	n/a	31

a Stakeholders were representatives of national or state agencies that administer CACFP or work with ECE to promote child nutrition and quality care.

b Sponsors were public or private non-profit organizations that take on administrative responsibilities of operating CACFP on behalf of ECE programs.

c ECE directors included directors of CACFP and non-CACFP center-based ECE programs.

d Interviews with stakeholders, sponsors, and ECE directors were conducted between March 2021 and September 2021. Preliminary results guided the development of the ECE director survey, which was administered after most interviews were completed, between July 2021 and November 2021.

ECE, early care and education; CACFP, the child and adult care food program; n/a, signifies not applicable.

### Data collection

Multiple methods (interviews, survey) were used for data collection. Given the broader focus on CACFP, data collection was guided by the Consolidated Framework for Implementation Research (CFIR) (20) that identifies contextual factors related to program characteristics, internal influences, and external influences that can influence program (CACFP) implementation.

Stakeholders, sponsors, and directors participated in interviews by telephone or video call (Zoom). Semi-structured discussion guides were developed for interviews with each respondent category, guided by prior studies of CACFP barriers/facilitators (21, 22), with input from partners at ECE agencies. The interviews probed into CACFP program characteristics, internal (e.g., implementation climate, structural characteristics, barriers/facilitators) and external factors (e.g., cross-agency efforts, external resources) that influence CACFP participation, and potential strategies to increase CACFP uptake. With a special focus on COVID-19, stakeholders and sponsors were asked about how COVID-19 had impacted their organization and work with ECE, and what supports/resources their organization provided to ECE to promote child nutrition and quality care during the pandemic. For ECE directors, interviews probed into how COVID-19 impacted day-to-day operations, including meals and the provision of child-care, and supports. Stakeholder and sponsor interviews lasted about 60 min, while director interviews lasted about 35 min. Participants who were able, without being in conflict with their organization's policy, received a thank you gift card. The interviews were conducted by trained members of the study team (TE, BJ). Stakeholders from 22 CACFP-associated agencies, representatives of 17 sponsor organizations, and 40 ECE directors participated in interviews.

To supplement director interviews, the study team administered an online survey using Qualtrics. Development of the survey was guided by preliminary results from the interviews with stakeholders, sponsors, and directors, with input from partners at ECE agencies. The director survey assessed barriers, facilitators, and potential strategies to promote CACFP participation by ECE programs. With a focus on the pandemic, the survey asked about the impact of COVID-19 on ECE programs' daily operations, menus and meals provided, and supports. The survey assessed demographic characteristics of participating ECE programs and directors and took about 10 min to complete. Participants received a thank you gift card. Overall, 100 directors completed the survey.

### Data analysis

Interviews were digitally-recorded and transcribed verbatim without identifiers. The transcripts were reviewed for accuracy and completeness, and imported into ATLAS.ti (version 3.4.5-2021-11, Berlin, Germany) for qualitative analysis. Members of the study team trained in qualitative analysis (MV, TE, BJ, and KL) reviewed the data and developed broad codes (themes) based on the discussion guides for the interviews and study aims. Separate codebooks were developed for stakeholders, sponsors, and directors. Within codes, contents were analyzed using a grounded theory approach described by Strauss et al. (23), after which they were categorized into emergent themes. Each transcript was initially coded by a primary coder, followed by a review by a secondary coder. Discrepancies in the application of thematic codes, were resolved by the secondary coder (TE, BJ, and KL). Because stakeholder, sponsor, and director interviews examined similar topics, and there were several areas of overlap in their responses, the study team pooled the interview data for the final summarization of results, and selected quotes that represented each theme. Survey data were analyzed in SAS (version 9.4, Cary, North Carolina) using frequencies and percentages.

## Results

Of 40 ECE directors who were interviewed, 58% (*n* = 23) participated in CACFP and 50% (*n* = 20) were in urban areas. Of 100 directors who completed surveys, 69% were in CACFP, while 66% in urban areas (Table 2). No demographic information was collected about stakeholders or sponsors.

**Table 2:** Demographic characteristics of center-based early care and education (ECE) programs from directors that completed a survey^a^.

	***n* = 100 ECE programs**
**ECE program characteristics**	***n*** **(%)**
**Location of ECE program**	
Rural	34 (34)
Urban	66 (66)
**Participation in CACFP**	
Yes	69 (69)
No	31 (31)
**For profit program**	
Yes	55 (55)
No	45 (45)
**Years of operation**	
< 1 year	1 (1)
1–5 years	16 (16)
6–10 years	9 (9)
More than 10 years	74 (74)
**Number of children enrolled at center**	
< 50 children	28 (28)
50–100 children	55 (55)
More than 100 children	17 (17)
**Recipient of child care subsidies**	
Yes	98 (98)
No	2 (2)
**Sources of meals served for lunch**	
Cooked on-site	76 (76)
Children bring their own food	15 (15)
Delivered meals (e.g., by a vendor or central kitchen)	7 (7)
Other (caterer, school district)	2 (2)

CACFP, the child and adult care food program; ECE, early care and education.

a Because of missing data, some frequencies may not add up to 100 ECE programs.

Six main themes emerged from the interviews, focusing on experiences pertaining to: the temporary closure of ECE programs; ECE providers' having to take on additional responsibilities and unanticipated expenses; challenges in keeping up with constantly changing COVID-19 guidance; difficulties with the shift from in-person to virtual trainings and CACFP compliance monitoring; changes to ECE nutrition practices; and prioritization of funding for ECE.

### Experiences during the temporary closure of several ECE programs

A common theme from participant interviews focused on their experiences during the temporary closure of ECE programs. Several directors described shutting down their programs temporarily because of fear/concern about the coronavirus, low child enrollment since many parents were working from home, and the occurrence of COVID-19 cases on-site. A director explained: “*When people were starting to get scared and everything happened, we had very low enrollment. I would only have 20–30 kids here. My staff was barely getting any hours… That's why we made the decision, when we did, to close.”* Some directors shared that they had to lay-off staff because of low child enrollment and the subsequent loss of income from tuition. Others explained that several ECE staff with concerns about COVID-19 opted not to return after their workplaces re-opened, resulting in staff shortages. The temporary closure of ECE also affected sponsors, resulting in the loss of their 15% fee for administering CACFP. A sponsor shared: “*We have more parents working from home, the whole COVID thing… That cut down on the enrollment in the centers, which of course, cut down on their reimbursement from the program (CACFP). That cut down on our administration funds… It's been a tough, tough year all round.”* On the survey, 80% of directors said COVID-19 adversely affected child enrollment, 62% said it led to revenue losses, and 49% reported losing several staff; 21% shut-down their program at some point (Table 3).

**Table 3:** COVID-19 experiences reported by center-based early care and education (ECE) program directors that completed a survey^a^.

	***n* = 100 ECE programs**
	***n*** **(%)**
**In what ways did COVID-19 affect your ECE program:**	
Made it difficult to find healthy food	30 (30)
Made it expensive to purchase healthy food	34 (34)
Decreased enrollment	80 (80)
Decreased revenue	62 (62)
Led to loss of several staff	49 (49)
Made it challenging to hire new staff	72 (72)
Made it difficult to find personal protective equipment	51 (51)
Made it expensive to purchase personal protective equipment	51 (51)
Center closed down	21 (21)
Other	9 (9)
**Since COVID-19, what kinds of changes, if any have you needed to make to your menus and meals provided:**	
We did not make any changes	38 (40)
Stopped serving family-style meals	39 (41)
Use more canned and frozen fruits and vegetables instead of fresh options	19 (20)
Cut down on food personnel staff	7 (7)
We've had to look for other sources of foods to cover cost of meals/snacks	6 (6)
We've had to rely more on fundraising	4 (4)
Stopped providing lunch or other meals	2 (2)
Stopped providing snacks	0 (0)
We've begun to consider joining CACFP	2 (2)
Other	5 (5)
**What kinds of supports did your ECE program receive during COVID-19:**	
More funding from our state agencies	51 (57)
Additional funding from other organizations	41 (46)
Access to information about where to find personal protective equipment	27 (30)
Being able to use the USDA waivers for CACFP	14 (16)
Trainings on how to make substitutions to meal patterns	6 (7)
Regular check-ins by external organization or sponsor	5 (6)
Information about where to purchase healthy food	3 (3)
Other	8 (9)

CACFP, child and adult care food program; USDA, U.S. department of agriculture; ECE, early care and education.

a Because of missing data and “select all that apply” response options, some frequencies may not add up to 100 ECE programs.

### Additional responsibilities and unanticipated expenses for ECE programs

ECE directors discussed taking on additional responsibilities in the form of protective health and safety measures, including conducting daily temperature checks, making sure that children kept on face masks, installing handwashing and sanitizing stations, and ensuring that children and staff washed and sanitized their hands. Other safety measures included not allowing parents into buildings, enforcing that staff wear face masks, and training staff on how to express emotion/praise for children behind their masks. A director said: “*I gotta make sure the kids have their mask on. I gotta take temps all day. I gotta make sure that, if they're coughing, and they're this and they're doing that, ok, I need to call mom to come pick them up…. This is a very stressful time for everybody right now.”* Several directors discussed incurring unanticipated expenses from purchasing personal protective equipment (e.g., gloves, masks) for their programs. Directors reported difficulty with finding personal protective equipment and supplies (e.g., toilet paper) early-on in the pandemic. While it is now easier to find protective equipment, many items have become more expensive, and some directors said the added cost was being transferred to families through tuition increases. A director shared: “*Our center has always… always had the gloves and most of the staff have personal masks… It's just that the cost for all of these things went way up… In response, we did have to up our pricing for the parents' stuff slightly.”* On the survey, over half of directors said COVID-19 made it difficult to find (51%) and purchase (51%) personal protective equipment, while 30% described community organizations as sharing information about where to purchase these items.

### Difficulty in keeping up with constantly changing COVID-19 guidance

Another theme from participant interviews focused on constantly changing COVID-19 guidance, especially in the early phase of the pandemic when little was known about the virus' transmission. Directors described difficulties in keeping up with constantly changing guidelines that were often inconsistent across federal, state, and local agencies, and lacking the support needed to implement protective regulations. A director shared: “*So, the confusion and the misinformation from the different agencies… the CDC (Centers for Disease Control and Prevention) saying 3 feet is okay. It doesn't mean it's ok for me because the state has to adopt that, the (state agency) has to adopt that, and then my county has to adopt that. So confusing information, and how do you tell parents everything that's going on.”* Stakeholders also shared challenges with keeping up with frequent changes to CACFP waivers, especially early in the pandemic when there was little guidance about how to implement new ECE requirements. A stakeholder shared: “*I think USDA could have given a whole lot more guidance. They put out all these waivers, but they didn't give any guidance as to information that they wanted us to gather until after the fact.”* After regulations began to ease and ECE programs started allowing on-site visits, a sponsor working with ECE programs across multiple states described challenges in keeping up with different mask mandates across states. A sponsor shared: “*That's a little bit challenging when you're a national sponsor, is, how do you say that this state don't have to wear masks in the center, but this state still does because licensing says we have to.”*

### Encounters during shifts from in-person to virtual training and monitoring

With a halt to on-site visits to ECE programs, stakeholders and sponsors shared their experiences of having to switch from conducting child nutrition trainings and CACFP compliance monitoring in-person, to using virtual platforms (e.g., Zoom). They discussed challenges stemming from lack of computers or internet access at some ECE programs, especially small and rural programs with limited financial resources. This restricted some stakeholders/sponsors' ability to conduct trainings and CACFP compliance monitoring virtually, instead having to rely on telephones. For some sponsors, the switch to virtual platforms prompted the need to invest in communication technologies (e.g., computers) and incur unplanned expenses, while also having to train staff in the use of technologies. A sponsor described: “*Everything we did was temporary. Then we realized that we need to invest in interactive computer systems that we could do training, and we could do monitoring, and that was huge expense at a time when we did not have money.”* Another challenge of virtual monitoring that was shared was the risk of sponsors missing out on detecting ECE nutrition practices that were not in compliance with CACFP. A sponsor explained: “*It's just my nature of being suspicious as a sponsor because I take the financial and administrative responsibility for them. I wonder, “do they have all those kids?”… That's something we can't see on the phone. We can see through a video, but it's just not the same.”*

### Changes to nutrition practices at ECE

Nutrition practices at ECE were impacted by COVID-19. Several directors described halting family-style meals. Children were socially-distanced and unable to sit together and interact at mealtimes. Some directors described switching to disposable utensils, while others opted for individually-packaged meals to prevent transmission of the coronavirus. A director said: “*So, we have made no changes to breakfast or snack. Like I said, the vendors switched from family style meals to individually-packaged… I just wish we could all sit around a table together again, but we cannot.”* Directors reported food shortages, especially of milk, early in the pandemic, but CACFP waivers that allowed programs to make food substitutions were described as helpful. A director said: “*I mean, thank God that we had meal waivers that we could file…. We were limited to the amount of milk and bread that we could purchase, for one thing. We were allowed to apply for waivers to substitute… Purchasing milk, it wasn't that big of an issue… but purchasing the right kind of milk was.”* In general, participants described the CACFP waivers as beneficial to ECE. Some stakeholders and sponsors recommended that aspects of the waivers be made permanent, in particular noting the benefit the waiver for non-congregate meals would have for programs that might have to shut down for weather-related reasons or emergencies. They also described how the waiver for virtual monitoring would save time and cost of travel to rural/remote areas by sponsors. For stakeholders/sponsors who recommended not making the waivers permanent, the need to switch back to in-person compliance monitoring was discussed as a way to encourage in-person interactions between stakeholders, sponsors, and directors, which they perceived would signify a return to normalcy. On the survey, nearly one-third of directors said COVID-19 made it difficult to find (30%) and more expensive to purchase (34%) healthy foods. Forty-one percent (41%) stopped serving family-style meals, and 20% used more of canned/frozen fruits and vegetables instead of fresh options, while 40% did not make changes to menus/meals provided.

### Need to prioritize ECE funding

Despite the challenges, participants shared that a positive outcome of the pandemic is that it drew attention to ECE's essential nature, and highlighted the need to prioritize allocation of federal and state funding to support ECE. A director said: “*We get left out of conversations about teachers. We get left out of conversations about essential workers, and I think our whole industry, and this just brought it to the forefront.”* Directors reiterated that emergency funding from state agencies, community organizations, and parent donations helped to keep programs afloat. A director said: “*(State agency) provided a grant for us. And then, of course, the PPE (personal protective equipment) grant. There's been quite a few organizations that have helped us out.”* Describing parental contributions, a director said: “*We've had silent auctions to raise money. We have a lot of generous parents that have donated things, so that's how we've stayed afloat.”* Describing the need for continuous funding for ECE after the pandemic ends, a stakeholder said: “*With COVID, I feel like we're getting a lot of funds, desperately needed, but I worry we're gonna get all these funds, and then after we're over this pandemic hump, they go, “Okay, you all don't need that anymore”, and we're like, “no, no, no, we've always needed that”.”* On the survey, 57% of directors reported relief funds from state agencies, and 46% reported funding from other organizations as supports received during the pandemic.

## Discussion

This paper describes experiences of stakeholders, sponsors, and center-based ECE program directors pertaining to child nutrition and the provision of child-care during the COVID-19 pandemic. Several areas of overlap were noted with regards to responses from stakeholders, sponsors, and directors. Themes from the interviews illustrated participants' experiences when several ECE programs had to shut-down temporarily, take on additional responsibilities and unanticipated expenses, and comply with constantly changing COVID-19 guidance from government agencies. Additional themes focused on participants' experiences during the shift from in-person to virtual trainings and compliance monitoring for CACFP, the pandemic's impact on nutrition practices at ECE, and the increased focus on the need to prioritize funding for ECE. Results from the interviews were consistent with director reports on the survey. While other studies have reported some of these experiences and related challenges during COVID-19 (7, 24–27), it is worth noting that Quinn et al. (26), found that a majority ECE staff (62%) described themselves as being “proud or grateful” for their designated roles as essential workers during the pandemic.

A notable strength of this multi-method study is that it provides insight into the challenges and supports for ECE providers during COVID-19. While directors discussed stressors associated with taking on measures to protect the health of their staff and enrolled children/families, they also highlighted benefits of funding received from government agencies, community organizations, and parent donations and fundraising. Funding was critical to how well ECE fared during COVID-19 (24). Head Start, a federally-funded program, was better equipped to use CACFP to provide nutritious meals to children (24). However, for programs relying on tuition from families, the added cost of shifting food preparation and food distribution to models not covered by CACFP and purchasing personal protective equipment posed challenges (24). Nevertheless, as indicated by study participants, a positive outcome of COVID-19 is that it brought ECE's essential nature to the forefront and highlighted the need to prioritize funding for ECE. The federal government passed legislation to make funds available to states to support ECE (e.g., CARES Act) (28), which were used in various ways, including to support ECE programs that provided child care to essential workers, and incentivize programs to stay open during periods of low child attendance (28).

Additional challenges reported by study participants centered around keeping up with constantly changing COVID-19 guidance with little or no implementation supports provided; conducting trainings and CACFP compliance monitoring virtually with staff at stakeholder/sponsor agencies who were not technology-savvy and ECE programs without computers/internet access; and navigating food shortages early in the pandemic. Nevertheless, participants described the CACFP waivers as beneficial. While some recommended making aspects of the waivers permanent, others cited the need to discontinue the waivers as a way to signify a return to normalcy. The waivers helped to ensure that children at-risk for food insecurity would receive nutritious meals daily, even if their ECE programs were closed (7, 8, 29). Reinforcing benefits of the waivers, Stephens et al. (6) found that compared to non-CACFP programs, ECE programs in CACFP were more likely to offer approaches, such as home meal delivery, grab and go meals, and distribution of food boxes to connect children/families with healthy foods during the pandemic. However, use of the waivers by ECE programs were not without challenges, including the availability of limited ECE staff to assist with meal preparation/distribution, difficulty in verifying child participation in CACFP during parental pick-up of meals, and the challenges of ECE programs with adapting to technology for virtual compliance monitoring (29). While the waivers have been extended through June 30, 2023 for home-based ECE programs, individual states have to put in formal requests to USDA if they want similar extensions (30). Unfortunately, COVID-19 forced many ECE programs to shut-down permanently or stop participating in CACFP altogether, as evidenced by the 22% decline in center-based ECE programs in CACFP from March-September 2020 vs. the same period in 2019 (31).

This study has limitations, including the relatively small sample of participants that prevented analysis by state or CACFP participation, which in turn may limit generalizability of the results. Interviews and surveys were administered in English. While development of the director survey was guided by preliminary results from the interviews and input from ECE partners, the survey was not tested for validity/reliability. A strength is the inclusion of stakeholders, sponsors, and directors' perspectives. Additionally, participants were recruited from four states, with a mix of urban/rural programs, and CACFP/non-CACFP programs. Our sampling design was based on purposive recruitment across subgroups (stakeholders/sponsors/directors) and states. To provide insight into potential thematic saturation, coders examined the number of times themes and high frequency issues were mentioned across subgroups, until there was consensus that the same key themes were emerging repeatedly. The study findings highlight the need for ongoing interventions, including investments in new communication technologies for ECE, provision of trainings in the use of technology, and creation of blueprints by governmental agencies to provide stakeholders/sponsors/directors with strategies for navigating similar pandemics or other emergency events in the future. Ongoing policy supports would be helpful, including federal funding for ECE and flexibility with CACFP waivers so they can be used as needed to ensure that ECE programs are prepared for future pandemics and enhance their efforts to promote child nutrition. Now that states have eased up on the protective guidance that were put in place during the initial phase of the pandemic, it would be important to understand what kinds of health/safety measures that individual ECE programs have put in place to protect children/staff. Studies are needed to quantify proportions of ECE programs nationally that shut-down permanently due to COVID-19 and assess the impact on ECE access. Efforts to increase CACFP uptake, which varies widely across states, would reduce poverty-related health disparities among underserved children.

## Data availability statement

The original contributions presented in the study are included in the article/supplementary material, further inquiries can be directed to the corresponding author.

## Ethics statement

The studies involving human participants were reviewed and approved by Indiana University Bloomington and the University of North Carolina at Chapel Hill. Written informed consent for participation was not required for this study in accordance with the national legislation and the institutional requirements.

## Author contributions

TE was principal investigator, oversaw all aspects of the project, and led the development of this paper. BJ was project manager, participated in data collection and data analysis, and contributed to the development of this manuscript. KL was a research assistant, participated in data collection and data analysis, and contributed to the development of this manuscript. MV was the qualitative expert and contributed to data collection, data analysis, and manuscript development. DW was a senior project advisor and contributed to the conceptualization of the research project, project implementation, and the development of this manuscript. All authors contributed to the article and approved the submitted version.
